# Supplementary figures and images for: Altered hippocampal transcriptome dynamics following sleep deprivation
Source: Mol Brain. 2021 Aug 12;14:125. doi: 10.1186/s13041-021-00835-1 (PMC8361790; doi:10.1186/s13041-021-00835-1)

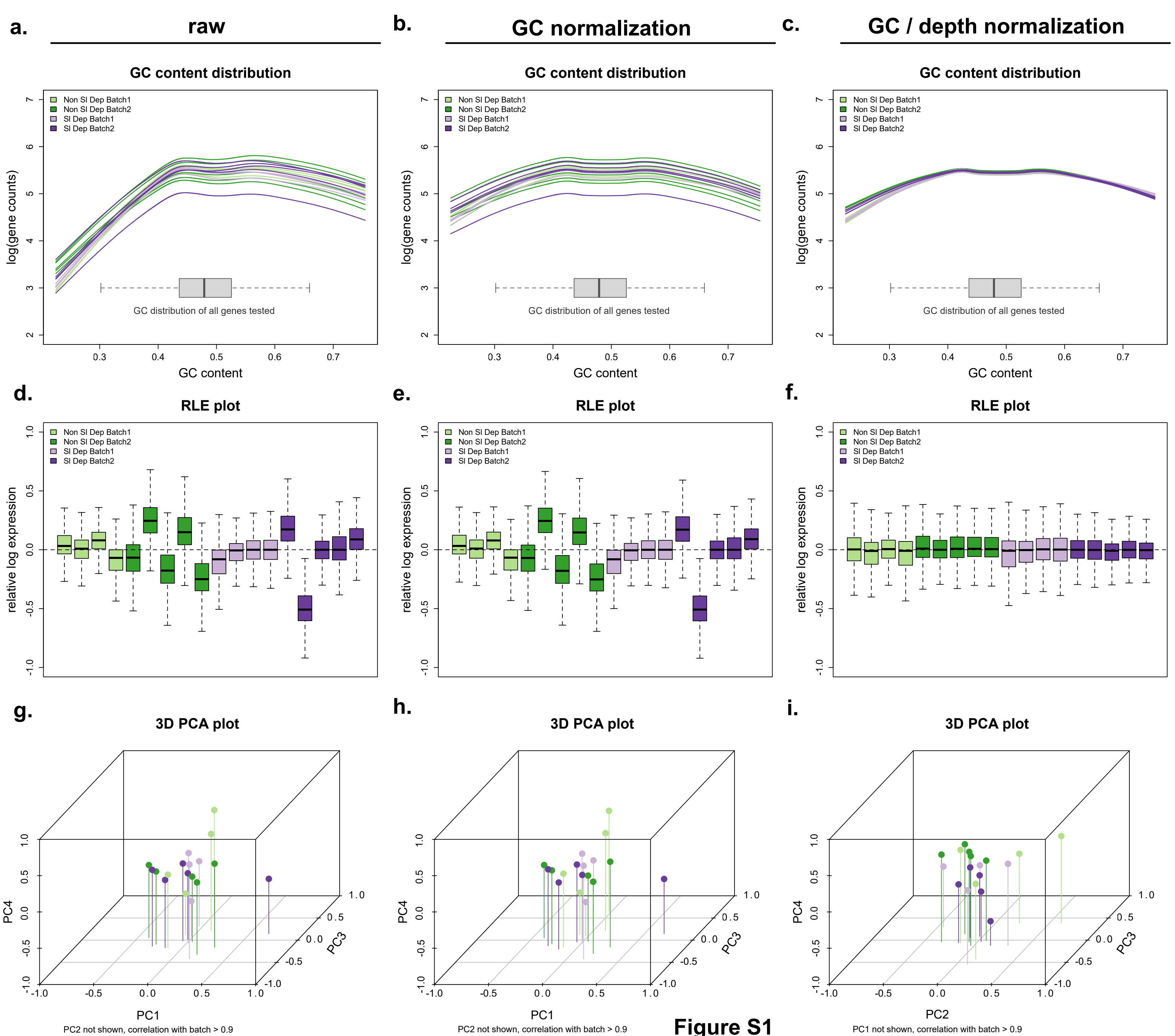

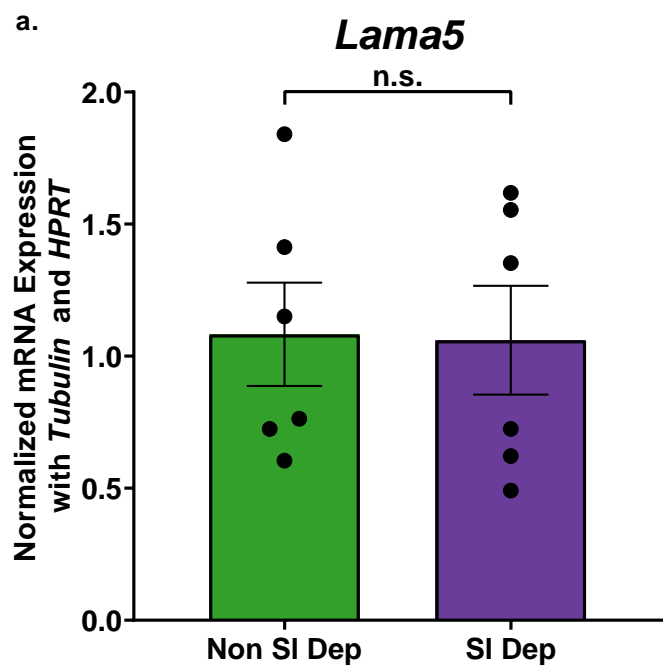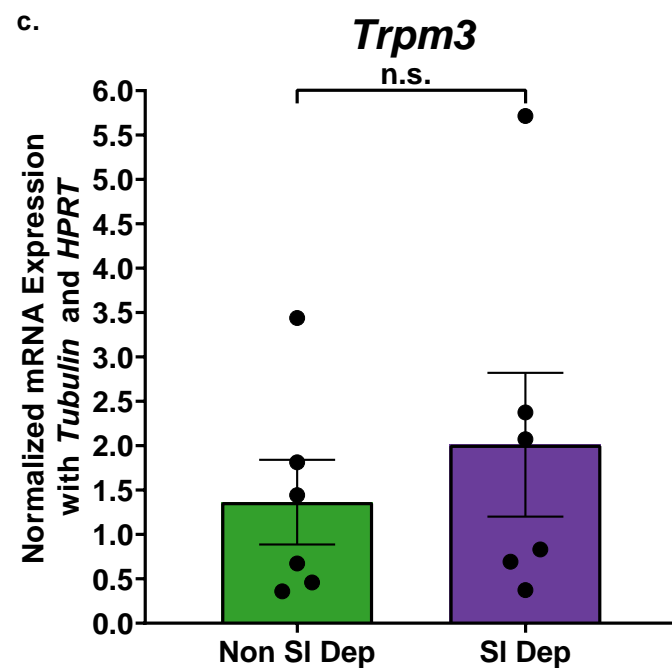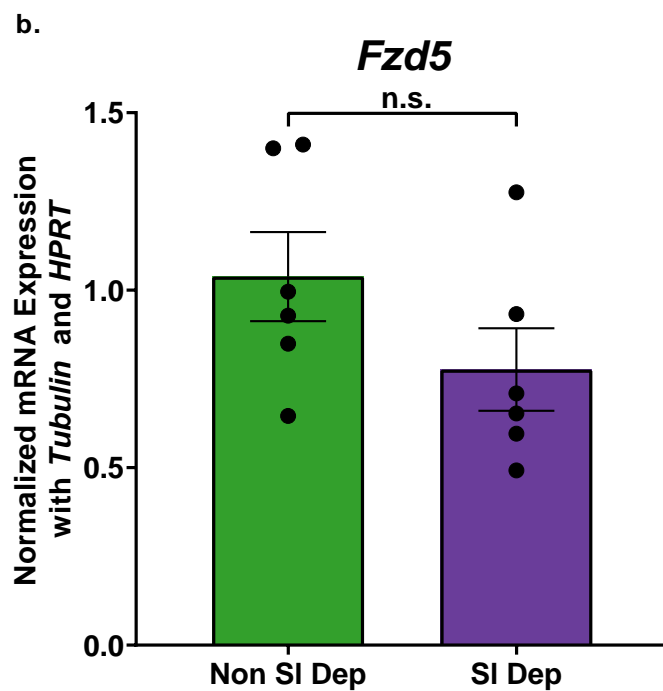

Figure S2

Supplement: Supplementary file 1 — Additional file 1: Figure S1. Normalization of RNA sequencing data. Distributional differences in GC content and variability in sequencing depth are sources of technical variability in RNA Sequencing data. GC content distributions (a) before normalization, (b) after full quantile GC content normalization, and (c) upper quartile sequencing depth normalization. Relative log expression (RLE) plots (d) before normalization, (e) after full quantile GC content normalization, and (f) upper quartile sequencing depth normalization. Principal component analysis (PCA) plots (g) before normalization, (h) after full quantile GC content normalization, and (i) upper quartile sequencing depth normalization. Figure S2. Validation of negative controls. From an independent cohort of mice (n = 6 in each group), RT-qPCR was used to validate the findings of negative control genes that showed no differential expression in the RNA sequencing results. (a) Lama5 (P-value = 0.9393), (b) Fzd5 (P-value = 0.1573), and (c) Trpm3 (P-value = 0.5076). Data are presented as mean ± SEM and normalized against two housekeeping genes (Tubulin and Hprt). Comparisons are evaluated using an unpaired t-test and n.s. denotes non-significant differences (P > 0.05). [file 13041_2021_835_MOESM1_ESM.pdf]
